# Supplementary material for: Unsupervised learning in probabilistic neural networks with multi-state metal-oxide memristive synapses
Source: Nat Commun. 2016 Sep 29;7:12611. doi: 10.1038/ncomms12611 (PMC5056401; doi:10.1038/ncomms12611)
Supplement: Supplementary Information — Supplementary Figures 1-15, Supplementary Tables 1-6, Supplementary Notes 1-11 and Supplementary References. [file ncomms12611-s1.pdf]

SUPPLEMENTARY MATERIAL

Supplementary Figures

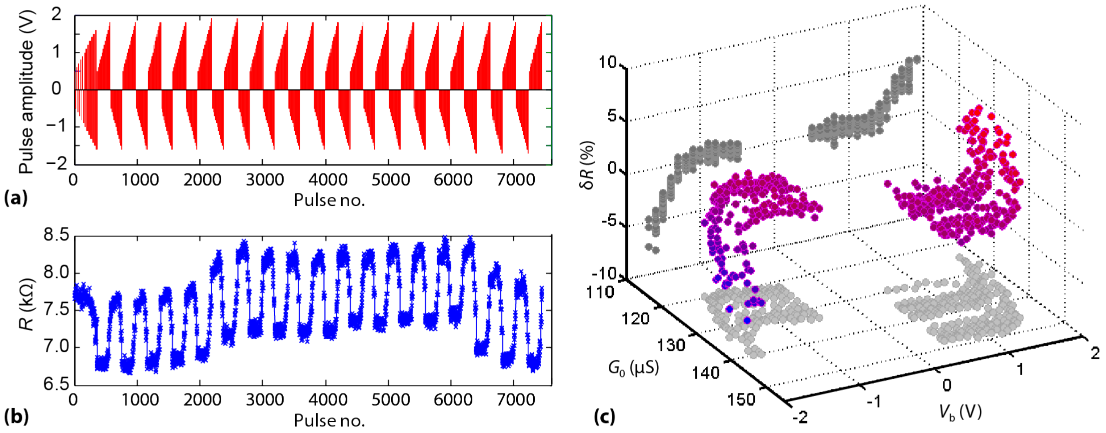

Supplementary Fig. 1: **TiO_2 devices support gradual switching.** (a) Biasing parameter optimiser testing protocol pulsing sequence as applied to subject device. (b) Resistive state time evolution in reaction to the stimulation from (a). (c) Change in resistive state as a function of applied pulse voltage (pulse duration 100 μs) and starting resistive state. Note that overall resistive state change is restricted to approximately ±15% around a baseline value (in this case approximately 130 μS). Dark gray projection shows resistive state change *versus* pulse voltage independent of starting resistive state.

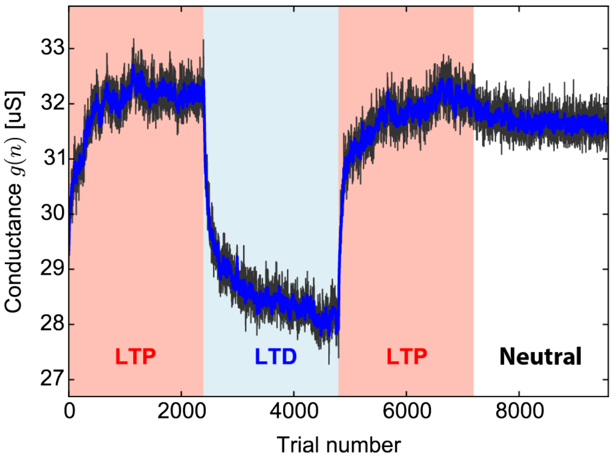

Supplementary Fig. 2: **The read operation used in this work was minimally invasive.** In the white, neutral region of the plot each pulse represents 5 ms at 300 mV. The read-out operation lasts about 20 ms at 200 mV by comparison. The device remains firmly at high conductance during this final stage of pulsing. Thus, in combination with Fig 1(d) we confirm that the read-out operation used in this work most likely does not affect DUT resistive state regardless of the conductance value at the time of read-out.

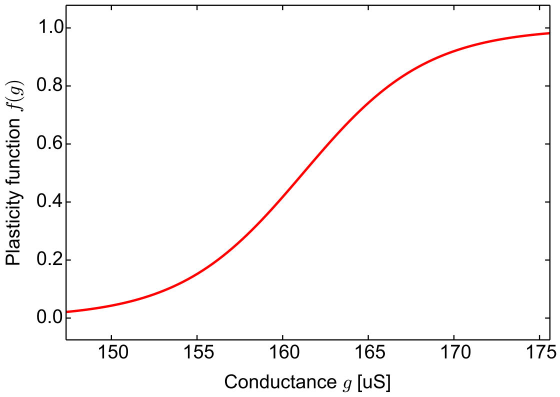

Supplementary Fig. 3: **Estimate of software plasticity from the fitted exponentials f^{LTP} and f^{LTD}.** A sigmoidal shape emerges.

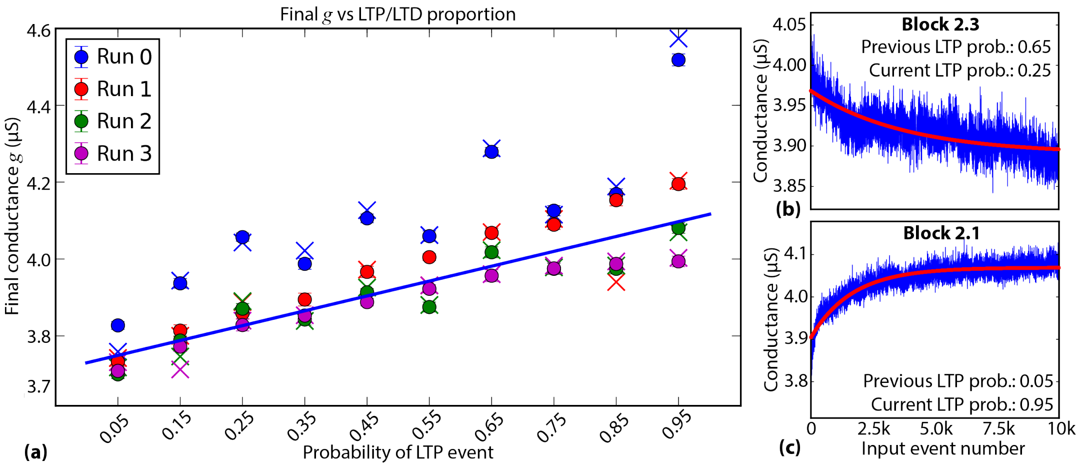

Supplementary Fig. 4: **Memristors encode conditional probabilities.** Same as Fig. 2 but extrapolated resistive state convergence points are now also shown as cross marks in panel (a). Points with the same colour at each LTP probability point arise from the same data block. Error bars: standard deviation, number of samples (individual resistive state readings) per data point $n = 25$. In (b,c) exponential fits are added to both data blocks.

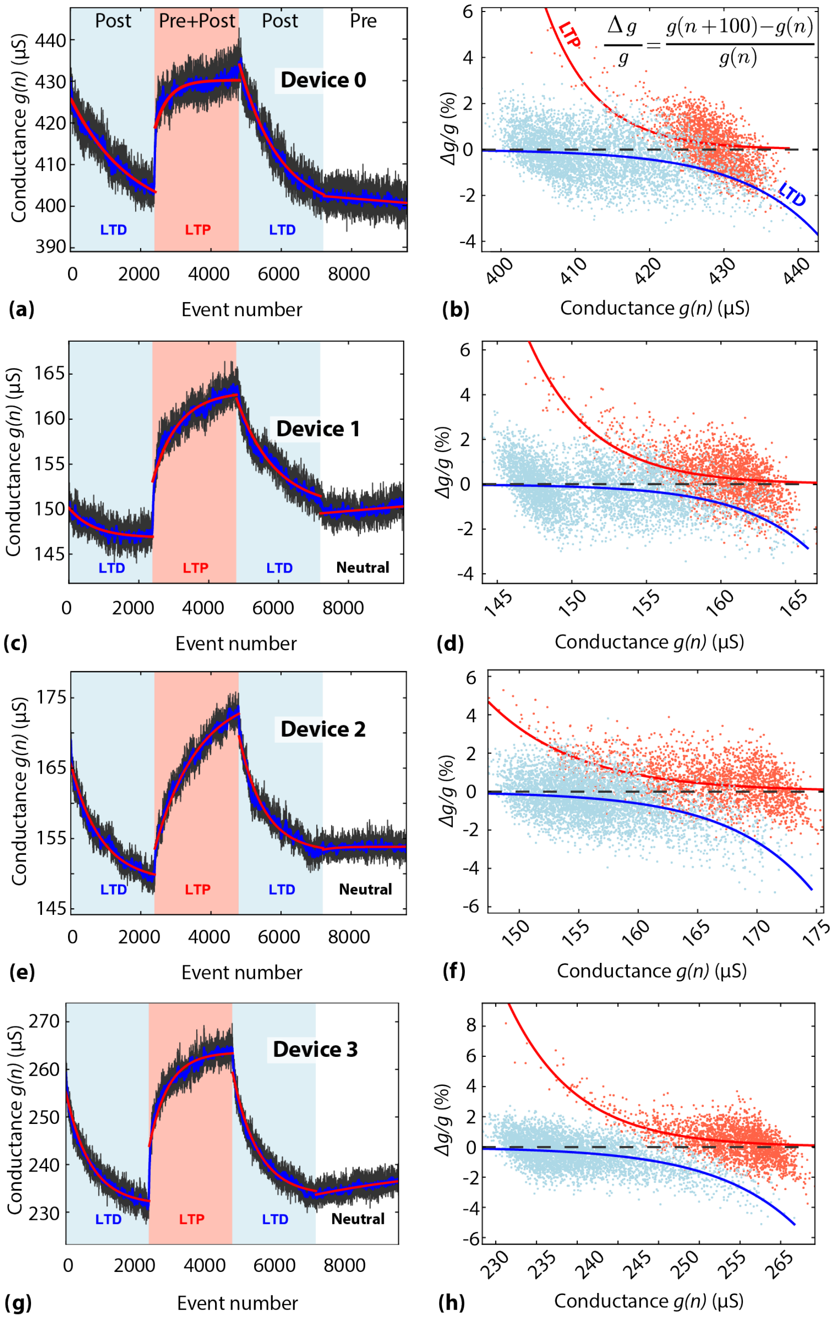

Supplementary Fig. 5: **Typical memristor behaviours under experimental protocol used for Supplementary Fig. 1.** Panels (a,c,e,g) and (b,d,f,h) as in Fig. 1(d,e) respectively for the four devices used as artificial synapses in this work.

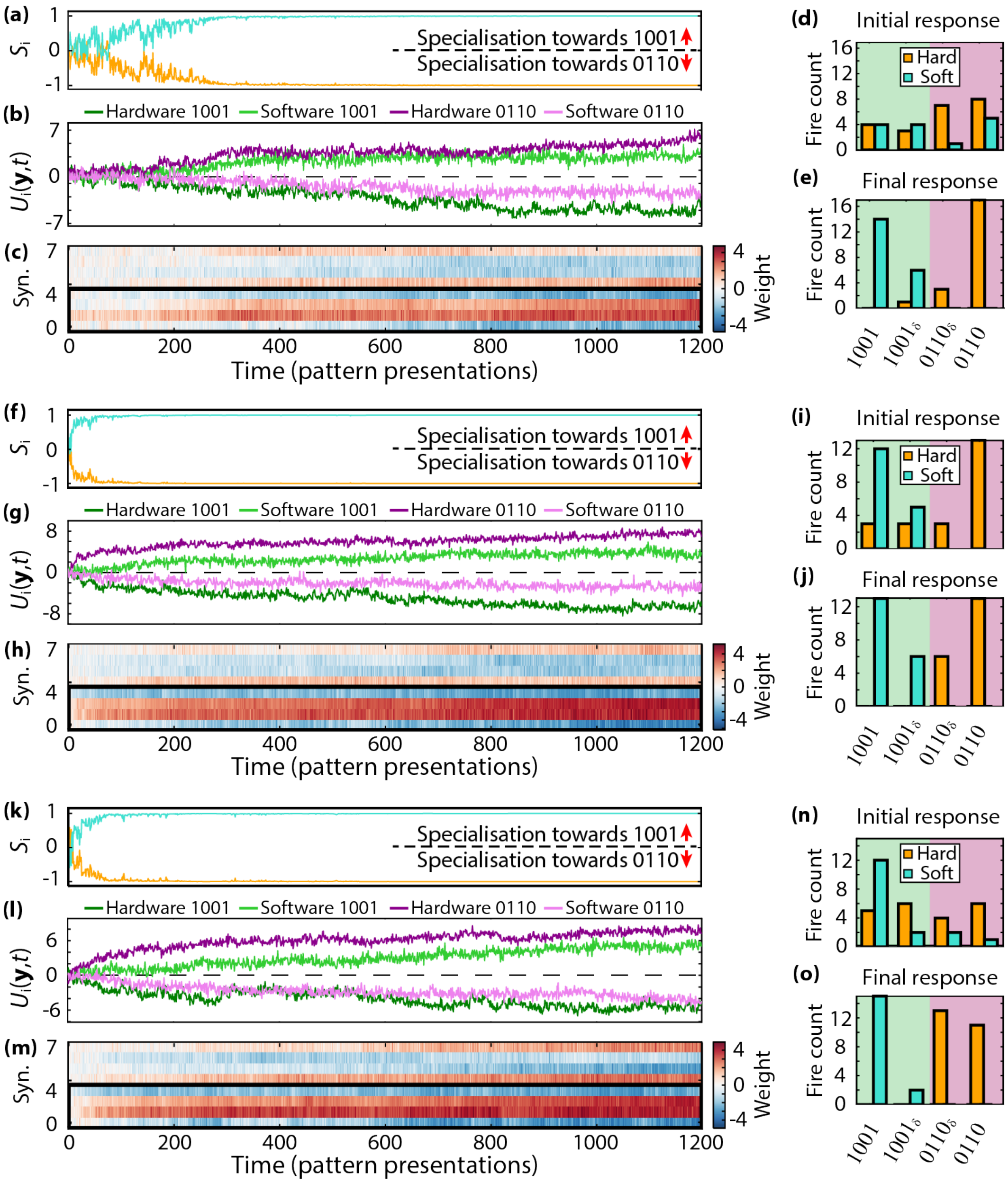

Supplementary Fig. 6: **Three consecutive runs of the ANN unsupervised learning experiment.** ((a-e), (f-j), (k-o)): as in Fig. 4. Before the beginning of each run all synapses are initialised so that no neuron has any preference for either prototype pattern. At the end of each run the prototype patterns have been segregated successfully. Legend similar to Fig. 4. The last run (k-o) corresponds to figure 3.

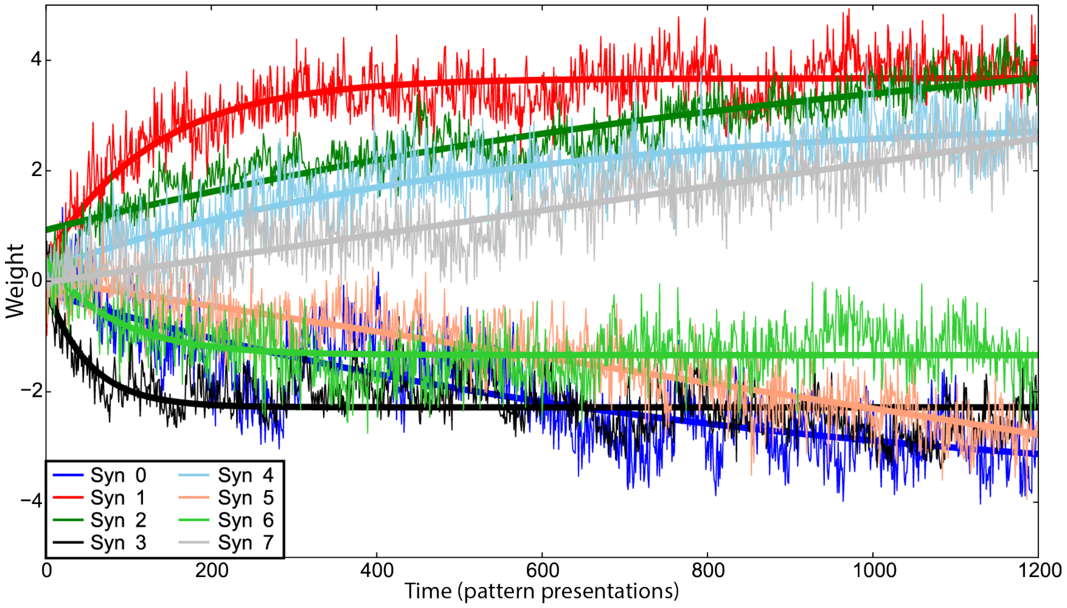

Supplementary Fig. 7: **Synapse behaviour during learning.** Evolution of hardware (synapses 0-3) and software (syn. 4-7) weights over the WTA network run from Fig. 3 (thin traces) and corresponding exponential fits (thick traces).

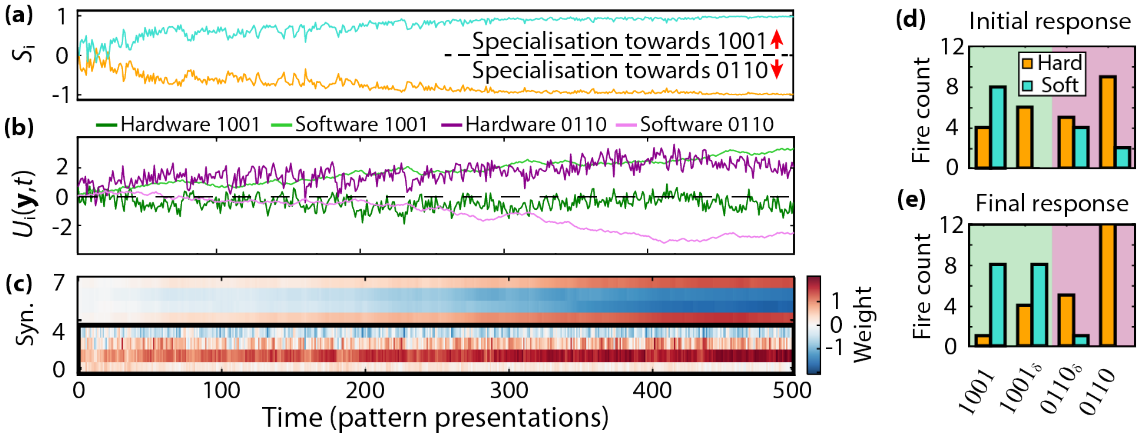

Supplementary Fig. 8: **Example of WTA learning in a test run where the software synapses are not afflicted by noise.** Panels (a-e) similar to Fig. 4.

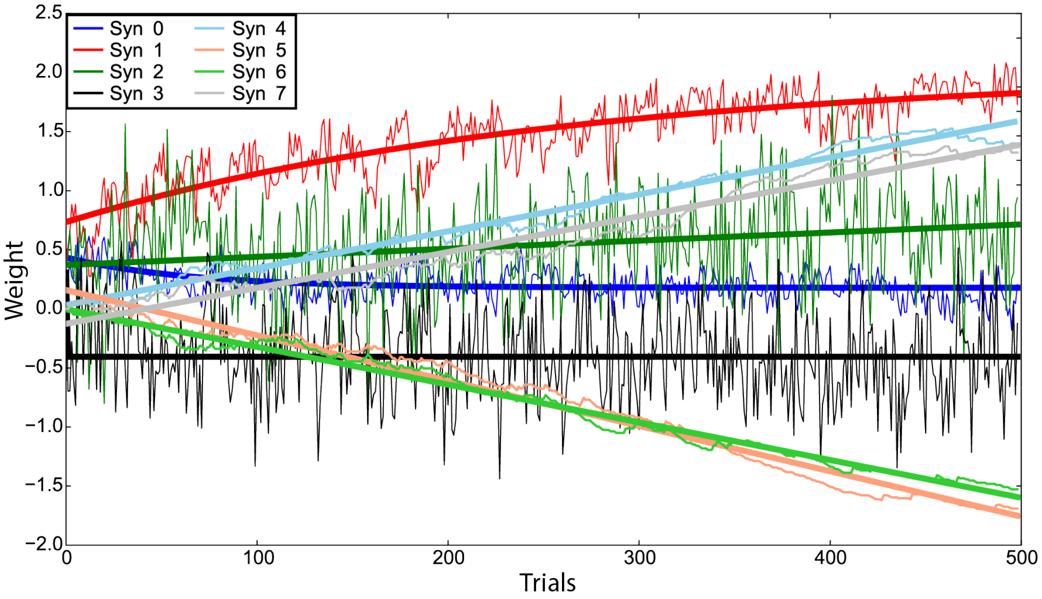

Supplementary Fig. 9: **Synapse behaviour during learning.** Evolution of hardware (synapses 0-3) and software (syn. 4-7) weights over the WTA network run from Supplementary Fig. 8 (thin traces) and corresponding exponential fits (thick traces).

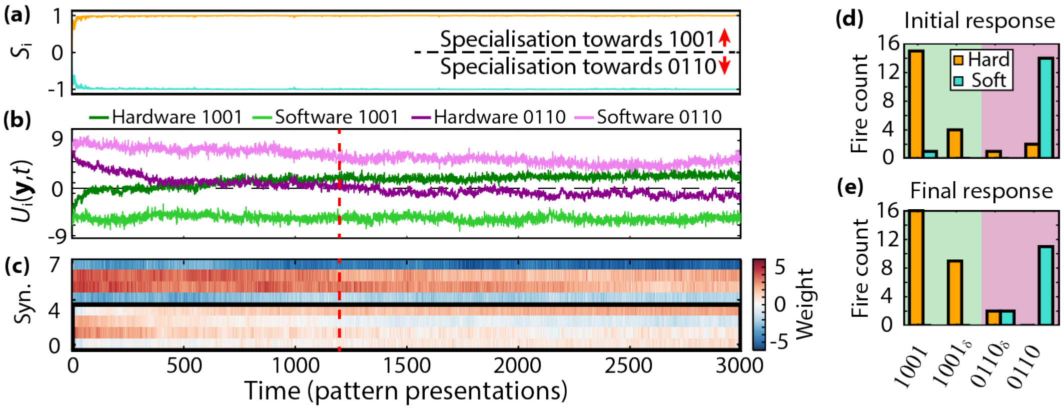

Supplementary Fig. 10: **3000 trial learning reversal experiment immediately following the last trial from Fig. 4.** Panels (a-e) similar to Fig. 4. The 1200 trial point is marked by the red, vertical, dashed lines in (b,c). The hardware synapses successfully switch their preference to pattern 1001 by the end of the run as evidenced by the computed membrane potentials shown in (b). At the 1200 trial mark this hasn't yet occurred very clearly.

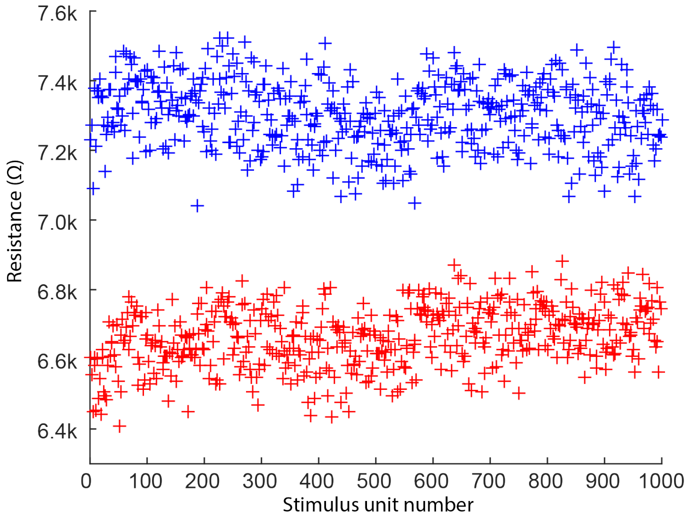

Supplementary Fig. 11: **Endurance data from TiO_2-based device family used for this work.** 500 full cycles of stimulate-assess are shown in the figure.

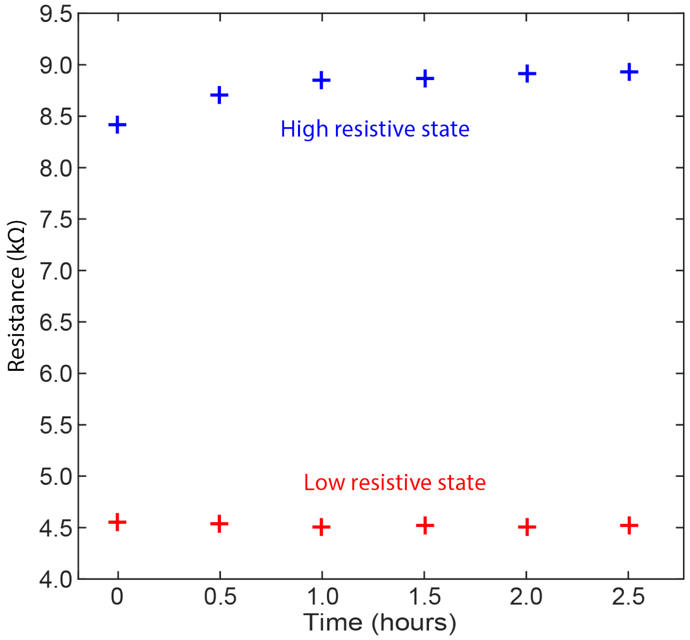

Supplementary Fig. 12: **Retention data from TiO_2-based device family used for this work.** Both high and low resistive states were individually checked for drift.

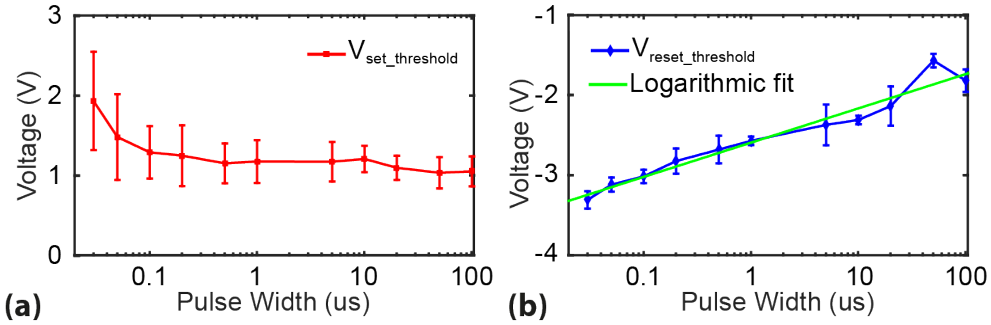

Supplementary Fig. 13: **Voltage-time dilemma in TiO_2-based memristors.** Estimated threshold voltages for LTP-like SET resistive state transitions (a) and for LTD-like RESET transitions (b) are shown as a function of applied pulse durations. In the case of RESET transitions we see a good exponential fit, whilst in the case of SET transitions the relation grows faster-than-exponentially. Standard deviation bars shown, number of samples (individual resistive state readings) per data point $n = 17$. Data presented previously by our group in reference [1].

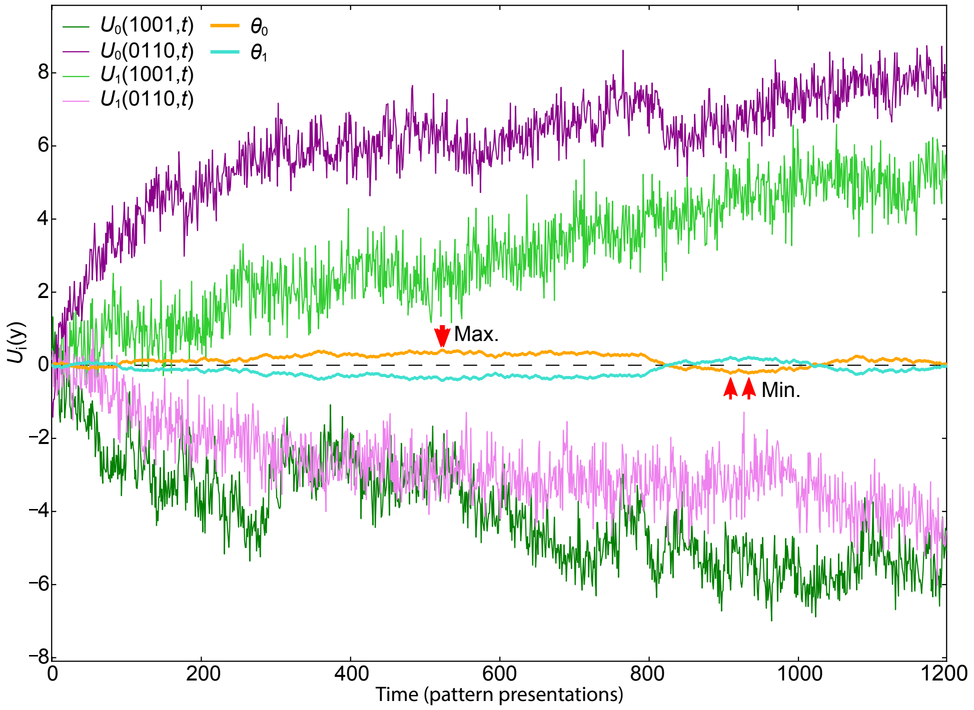

Supplementary Fig. 14: **Membrane potentials and homoeostasis during learning.** Computed full membrane potentials U_i(pattern, time) for both hardware ($i = 0$) and software ($i = 1$) neurons to patterns 0110 (purple) and 1001 (green) including the influence of the homoeostatic term θ_i from eq. (3); same as Fig. 3(c). Additionally, the contribution of the homoeostatic plasticity to the membrane potential is also plotted alone in orange/cyan. Red arrows indicate the trials when the homoeostatic correction term reaches its maximum (+0.419) and minimum (-0.225) values.

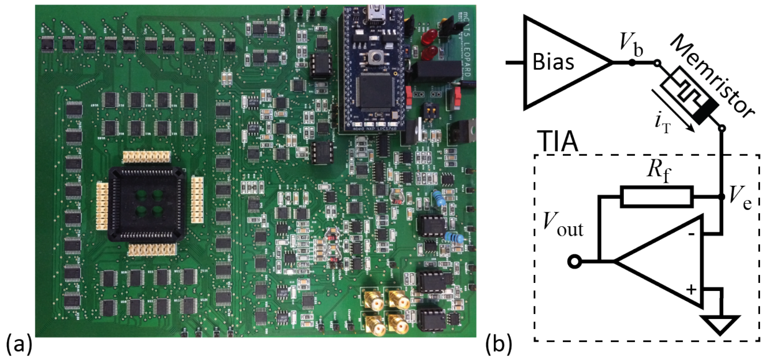

Supplementary Fig. 15: **Memristor characterisation and handling instrumentation.** (a) Photograph of and (b) read-out scheme used by the instrumentation used to carry our all experiments in this work.

Supplementary tables

Supplementary Table 1: Drift in memristor resistive state as a result of the application of only pre-type events.

DUT ID	Resistive state drift (μS)	% of resistive state range (abs. val.)
0	-1.65	5.15
1	+0.79	6.05
2	+0.40	1.76
3	+2.83	9.42
Read-check	+0.31	-

Case Read-check corresponds to Supplementary Fig. 2; all other cases directly from Supplementary Fig. 5. The resistive state range is directly computed from Supplementary Table 3 as the difference between the conductance levels corresponding to the weight values $[+2.2\,V, -2.2\,V]$ (final - initial). The read-check case has no defined operating range.

Supplementary Table 2: Voltage threshold levels extracted under 100 μs pulsed stimulation for the devices used in this work.

Device ID	$V_{\text{th, LTD}}$ (V)	$V_{\text{th,LTP}}$ (V)
0	-1.07	+0.98
1	-1.08	+0.91
2	+1.31	-1.19
3	-1.40	+1.19

Supplementary Table 3: Biasing parameters and conductance-to-weight mappings used for all WTA network runs.

**Device ID**	**LTD ampl. (V)**	**LTP ampl. (V)**	**Conduct. at weight = -2.2 (μS)**	**Conduct. at weight = +2.2 (μS)**
0	-0.90	+0.90	398	430
1	-0.90	+0.90	150	163
2	+1.20	-1.10	153	176
3	-1.30	+1.10	234	264

Supplementary Table 4: Order of test blocks in each conditional probability encoding experiment run - see Fig. 2.

**Run no.**	LTP probability (%)									
	**0.05**	**0.15**	**0.25**	**0.35**	**0.45**	**0.55**	**0.65**	**0.75**	**0.85**	**0.95**
0	7th	5th	3rd	6th	4th	8th	2nd	10th	9th	1st
1	10th	9th	8th	7th	6th	5th	4th	3rd	2nd	1st
2	7th	5th	3rd	6th	4th	8th	2d	10th	9th	1st
3	10th	9th	8th	7th	6th	5th	4th	3rd	2nd	1st

Supplementary Table 5: Initial and final weights for software and hardware synapses for WTA network runs 1 (Fig. 3) and 2 and 3 (Fig. 4).

**Run no.**	**Synapse type**	**Initial/final**	**Synapse ID**			
			1	2	3	4
1	Memristive	Initial	0.046	-0.423	-0.578	-0.414
		Final	-2.823	4.088	3.188	-2.269
	Software	Initial	0.064	0.228	-0.069	0.381
		Final	2.471	-2.542	-1.969	2.626
2	Memristive	Initial	-3.400	3.862	3.528	-2.362
		Final	1.909	-0.101	0.006	1.705
	Software	Initial	-2.734	4.465	3.286	-2.922
		Final	-3.322	3.614	2.982	-3.964
3	Memristive	Initial	1.614	0.263	0.348	1.549
		Final	-2.600	4.267	3.106	-2.675
	Software	Initial	2.179	-1.092	-0.697	2.859
		Final	2.926	-2.100	-2.167	1.886

Supplementary Table 6: Noise quantification for weight evolution during WTA network run from Fig. 3.

Synapse ID	$\Delta w_{\mathrm{total}}$	$\sigma_{\mathrm{residual}}$	$\sigma_{\mathrm{measerror}}$	$\sigma_{\mathrm{unexplained}}$
0	-2.9205	0.6062	0.3673	0.4823
1	3.3125	0.4167	0.3539	0.2200
2	2.7363	0.3470	0.2002	0.2834
3	-2.1748	0.4364	0.2534	0.3553
4	2.4748	0.4236	-	-
5	-2.7820	0.4152	-	-
6	-1.7032	0.4580	-	-
7	2.5998	0.4329	-	-

$\sigma_{\mathrm{residual}}$: Standard deviation of residuals of weight *versus* exponential fit from Supplementary Fig. 7. $\Delta w_{\mathrm{total}}$: Overall change in weight over the duration of the learning run as extracted from the polynomial fitting (final - initial). σ_{meas}: Uncertainty directly attributable to measurement error. $\sigma_{\mathrm{unexplained}}$: Remaining uncertainty. All values in units of abstract weight.

Supplementary note 1

Device characterisation and behaviour. The capability of TiO_2-based memristors to encode conditional probabilities largely relies on their ability to support gradual switching. Supplementary Fig. 1 shows the biasing parameter optimiser test routine [2] being applied on a single device. During this routine the device under test (DUT) is subjected to a series of pulse trains in alternating polarities. Each pulse train consists of a succession of progressively higher voltage pulses; all at fixed duration (Supplementary Fig. 1(a)). The effect of each voltage amplitude used on DUT resistive state is assessed by measuring resistive state between pulses. The test shows how the choice of bias voltage determines the speed of switching (Supplementary Figure 1(c)). We find that in our devices appropriate choice of pulsing voltage can lead to gradual switching corresponding to very small δR in response to input stimulation.

Applying successive barrages of identical, pulsed stimuli (LTP only or LTD only) as described in Fig. 1 confirms the capability of gradual switching and uncovers the dependence of the magnitude of switching on the value of the running conductance. Supplementary Fig. 5 shows results from the experimental procedure carried out in Fig. 1 on all devices used for this work. We note that all devices are well-behaved, with LTP and LTD easily fitting to the exponential model used in Fig. 1.

Moreover, Supplementary Fig. 5(e) shows a typical case of cycle-to-cycle variation in memristive devices. Final resistive state at the end of the second LTD event block is slightly different compared to the first LTD block. Whilst this may be at least partially explained by incomplete convergence to an equilibrium point, our experience with the TiO_2-based devices suggests that cycle-to-cycle variation is likely to play a role in this phenomenon.

Another important aspect of device behaviour is the voltage-time dilemma, that is the trade-off between pulse duration and voltage. We tested our samples with the biasing parameter optimiser routine at different pulse widths and recorded the pulse voltages at which the resistive state of the DUT had changed by 2% *versus* its state at the start of the test. The obtained values provide rough, but comparably obtained estimates of the DUT voltage threshold. Supplementary Fig. 13 shows extracted threshold voltages from a typical device in the same family as used in this work *versus* pulse duration whilst Supplementary Table 2 summarises the 100 μs pulse thresholds extracted for the devices used in this work. The exponential relation between pulse duration and pulse voltage is encouraging towards the notion that switching can be achieved at significantly lower power cost if shorter, but stronger pulses are used as stimulation.

The thresholded nature of switching in our devices as shown in Supplementary Fig. 1 provided good read-disturb immunity to our devices. Fig. 1(d) shows that the DUT read-out operation did not lead to appreciable changes in DUT resistive state when the DUT is at its minimum operational conductance. We ran experiments to confirm that this is still the case when the DUT is at its maximum operational conductance. Results are shown in Supplementary Fig. 2, confirming the immunity of our devices to read-disturb at both extremes of their operating resistive state range. In addition, we

quantified these results by fitting conductance evolution data from the neutral regions (pre-type stimulation only) of Supplementary Figs 5(a,c,e,g) and 2 to exponentials via least-squares optimisation and then computing the fitted change in conductance at the start *versus* the end of the region. The results, summarised in Supplementary Table 1, indicate that the effect is small (less than 10% of DUT resistive state range as defined in Supplementary Table 3).

Finally, basic endurance and retention data is shown in Supplementary Figs 11, 12. The endurance run was conducted by repeatedly applying stimulus units (trains of 10 identical pulses lasting 100 µs at +1 V or −1 V amplitude) of alternating polarities, each followed by resistive state assessments (1 assessment = average of 5 reads). Results indicate reliable and repeatable switching of our TiO_2 devices for 500 cycles (that is 1000 stimulus units) with a small but clear (approximately 3% of Low Resistive State (LRS) resistive state level) window between High Resistive State (HRS) and LRS. The retention run was carried out by driving a test device at its operational resistive state ceiling, measuring resistive state for 2.5 hours in 30 minute intervals and then driving the device to its operating resistive state floor and taking another set of half-hourly resistive state measurements. We notice that the low resistive state is very stable (max. - min. value: approximately 44 Ω) whilst the high resistive state experiences a slight upward drift (max. - min.: approximately 505 Ω corresponding to approximately 13% of the resistive state operating range of approximately 4 kΩ).

Supplementary note 2

Functional form of plasticity. The estimated functional form for software plasticity is shown in Supplementary Fig. 3. This relies on the two exponential fits for f^{LTP} and f^{LTD} from Fig. 1(e).

Supplementary note 3

Experimental protocols. In the experiment testing for the capability of memristors to encode conditional probabilities, four test runs were carried out. Two of them used test blocks visiting the LTP probability points in scrambled order for the purposes of confirming that results obtained from the other two runs were not a consequence of visiting the various LTP probability points in a monotonically decreasing order. The precise sequence in which LTP probability points were visited are shown in Supplementary Table 4.

In all WTA network experiments both the biasing parameters used to implement plasticity and the mappings between memristor conductance and synaptic weight were kept constant. The numbers used are summarised in Supplementary Table 3. The initial and final software and hardware weights for each WTA network run are summarised in Supplementary Table 5.

The effects of homoeostasis can be observed by examining the computed membrane potential response of the hardware-synapse neuron for the two prototype patterns

and noting how significant the effect of the homoeostatic term is. This is shown in Supplementary Fig. 14 for the ANN run corresponding to Fig. 3, where the homoeostatic term fluctuated between $+0.419$ and -0.225 units of abstr. weight. However, the homoeostatic term can take much larger values, reaching a magnitude maximum at -1.333 abstract weight units during the learning reversibility check ANN run, which indicates a potentially powerful effect on overall membrane potential.

Supplementary note 4

Fitting converged conductance *versus* LTP/LTD composition. The linear fitting used for Fig. 2(a) followed the formula:

$$S(p) = a \cdot p + b \tag{14}$$

where $S(p)$ is final, converged conductance as a function of LTP/LTD composition p, $a = 3.87 \cdot 10^{-7}$ and $b = 3.73 \cdot 10^{-6}$).

Supplementary note 5

Quantifying quality of convergence. The quality of convergence achieved during the experimental runs shown in Fig. 2(a) is very hard to assess reliably given the difficulty in extrapolating how memristors might behave after the end of each 10^4-point data block. However, as a simple check the memristor resistive state evolution during each data block -conductance $g(k)$- was fitted to an exponential as per eq. 15. The constant offset term c then denotes the expected resistive state saturation level for each data block. Extrapolated convergence values are plotted in Supplementary Fig. 4 along with two data block runs and their corresponding exponential fits. We note that the exponential fits in both cases tend to qualitatively underestimate the degree with which the resistive state continues to drop/increase towards the end of each data block. Further study is required in order to understand precisely why this occurs and determine a more suitable fitting model. Moreover, we notice that on most occasions (38/40), despite the possible unsuitability of the exponential model as a fitting function, the extrapolated resistive state convergence points are within 400 nS of their counterparts as extracted from the experimental data. In the remaining two cases the conductance *versus* input event number plots does not exhibit a sufficiently strong saturating trajectory and causes the extrapolation to fail. We therefore conclude that: i) Incomplete convergence cannot be ruled out as a reason behind the qualitatively worse convergence observed for run no. 2, ii) preliminary checks attempting to fit data to exponentials do not lend support to this hypothesis but do not disprove it either and iii) exponential fits may be poor predictors of future memristor behaviour.

Supplementary note 6

Repeatability of learning. In order to demonstrate that the memristive synapses can repeatably perform learning as shown in Fig. 3, the experiment was performed three consecutive times. In each experiment run all devices were initialised through the memristor control instrument to values corresponding to an abstract weight of 0 (within the limits of the measurement noise). The software was then initialised to 0 weights (on top of which measurement noise was added during operation). The three experimental runs are shown in Supplementary Fig. 6 where we observe that the last run is the one from Fig. 3. In all cases the data clearly shows that both neurons start from a situation where they both display no specialisation on either pattern and simultaneously their membrane potentials show no inherent preference to either pattern. At the end of each run, the prototype patterns have been successfully segregated.

Supplementary note 7

Quantifying the weight evolution noise during WTA network runs. In order to quantify the noise present in the evolution of the memristive synapse weights throughout the WTA network trial shown in Fig. 3 we first fitted the weight data to first order exponentials of the form:

$$w(k) = a \cdot e^{-\frac{k}{b}} + c \tag{15}$$

where $w(k)$ the memristor synapse weight at input event k and a, b, c fitting parameters. Results are shown in Supplementary Fig. 7. The residuals were then extracted and their standard deviations computed. These results are summarised along with overall weight change throughout the WTA run Δw_{total} as estimated by the fittings in Supplementary Table 6.

It is important to note that the standard deviations of the residual levels computed will include contributions from at least three main components: First, The stochastic nature of the input signal. Second, in the case of the hardware synapses, random measurement error. Third, extra error introduced by the mismatch between the choice of fitting function and the underlying synaptic weight evolution dynamics. The random measurement error can be quantified by examining the standard deviation in the resistive state of the hardware synapses as computed from the neutral region seen in the left half of Supplementary Fig. 5 (residual *versus* exponential fitting to mitigate spontaneous drift effects). If we then combine the standard deviation in the resistive state with the mapping between resistive state and weight from Supplementary Table 3 we can compute the contribution of the measurement error to overall noise levels in units of abstract weight. These values are shown for the hardware synapses in Supplementary Table 6. Notably, software and hardware synapses show similar levels of overall noise. Note: throughout this analysis we have assumed that the distribution of residuals is normal. Whilst this may not be necessarily true, the overall values of standard deviation are still indicative of noise levels in the system.

Supplementary note 8

Comparison case: what if software synapses are immune to noise? For the purposes of comparison we have also carried out a WTA learning experiment where the software synapses were implemented without added noise. Results are seen in Supplementary Figs 8 and 9. The difference is very clear especially with regard to the progress of learning between the neuron using software and the neuron using hardware synapses (Supplementary Fig. 8(b)), but also when examining the evolution of synaptic weight.

Supplementary note 9

Learning reversibility timescale check. The learning experiments shown in Fig. 4 did not fully elucidate whether the system is truly capable of developing a new, stable weight configuration during reversal learning since the memristor synapses still exhibited notable changes by the end of the 1200 trial WTA run. For that reason, immediately after the conclusion of the experimental runs from Fig. 4 an additional reversibility run lasting 3000 trials was carried out. Results are shown in Supplementary Fig. 10 where we notice that after 1200 trials the system has not yet fully settled at a stable weight configuration. After 3000 trials, however, the reversal is very clear as indicated by the computed membrane potentials of the hardware neuron. Thus the system is truly capable of not just learning, but if necessary also complete relearning.

Supplementary note 10

Materials level interpretation. In this section we attempt to link the observations made throughout this paper to a materials-level interpretation on a working hypothesis basis, which is, however, not the focus of the current publication.

Pristine memristive Pt/TiO2/Pt devices used in this study being their lives at highly insulating states due to the stoichiometry of the oxide layer. The process of electroforming then serves to create a conductive path within the oxide, commonly called conductive filament (CF). During electroforming an external electric field is applied between the two electrodes oxygen vacancies and/or metal (titanium in our case) interstitials migrate towards the anode and accumulate until bridging the electrodes, consequently, reducing the pristine resistive state towards a LRS. It is now well known that the CF consists of oxygen vacancies in devices operating through valence change memory (VCM) mechanisms such as ours. Subsequent application of voltage in the opposite polarity (in systems exhibiting switching of the bipolar type) resets the device towards HRS by thinning, or breaking the CF. In this step, the oxygen ions fill some of oxygen vacancies cites disrupting the filament continuity, thus increasing the resistance the high resistive state (HRS) [3, 4, 5, 6]. It is worth mentioning, on one hand, that the pristine state is never recovered because of the influence of all the filament branches that where created during electroforming step, thus forcing the device to toggle between some LRS and HRS

resistance values far below the initial, pristine level. The stochastic nature of the CFs explains the variability in the threshold voltages; the voltage levels at which the device begin to experience switching towards lower (higher) resistive states. Notably, the precise magnitude of the voltage stimulus pulses affects the values of HRS and LRS between which the device can toggle: higher applied voltages enhance the HRS/LRS contrast, but at the expense of endurance and switching graduality (higher voltages - most of the resistive state change tends to occur upon the first pulse [7]). When applying long trains of constant voltage pulses the vacancies/ions susceptible to drift under the accumulated energy gradually migrate, resulting in a progressive shift in resistance until reaching a plateau (convergence), where no more vacancies/ions can drift unless the pulse amplitude or/and width are increased.

It is important to specify that especially when operating at near-threshold levels many pulses are needed to migrate all the vacancies/ions sensitive to the applied voltage. This is the basic explanation of the results depicted in Figure 2. The more LTP (LTD) events are applied to the device the higher (lower) the conductance becomes. At a probability of 0.05% of LTP events for example, the number of positive pulses overcomes the negative ones resulting in drifting more vacancies thus building the CF. The nature of the experiment in runs 2 and 4, which consists of applying LTP and LTD events to the device and slowly and regularly increasing (decreasing) the number of LTP (LTD) events at each event block, causes the final (and ideally converged) conductance to increase smoothly. However, larger variability in converged conductances was observed for run 1 and run 3, where the probabilities of LTP (LTD) events was randomly applied. These more abrupt changes in pulsing regime render the overall vacancy/ion drift more aggressive throughout each run and thus are the possible cause of the increased end result variability.

It is worth mentioning that the filamentary nature of the switching of our devices makes the ON state very stable, possibly because at that state the filament bridge is completely formed; determining whether this is indeed the case requires further study. However, the CF is disrupted and interrupted in the OFF state, and at the end of each pulse train the OFF resistive state drifts slightly, particularly immediately following stimulation interruption. This is observed in Supplementary Fig. 12 where the test device drifts from 8.4 kΩ to 8.9 kΩ within the first 30 minutes after stimulus interruption. The drift continued with smaller changes, from 8.9 kΩ to 9 kΩ for the following 2 hours, as can be seen. We attribute this to the active component of the resistive switching devices, named nano-battery effect [8]. Indeed, Valov et al. have studied this phenomenon and demonstrated that an inherent electromotive force (emf) exists within the device that causes the resistance value to change even when no external voltage is applied. This emf or diffusion is generated by the inhomogeneous charge distribution and charge motion resulting from the electroforming or set/reset processes. This happens at HRS where vacancy/ion drift occurs slowly, however, when a CF is completely formed, in the LRS, this phenomenon does not occur. The nanobattery effect is partially masked in this proof of principle study, as learning occurs under a constant barrage of input data, which allows the vacancies/ions to drift and achieve repeatedly relatively stable conductance values.

However, carefully studying the influence of this phenomenon in further exploiting this work should be considered. Interesting open questions for further research would be whether the presence of this emf materially affects the balance between potentiation and depression during network operation and to precisely what extent drift in resistive state after stimulation interruption is tolerable (even though results from Supplementary Figs 5 and 2 suggest the overall effect is relatively small).

Supplementary note 11

Measurement instrumentation. All experiments in this work were carried out using our in-house developed instrument shown in Supplementary Fig. 15(a) that derives from the work in [9]. The instrument uses a trans-impedance amplifier-based (TIA) read-out procedure which is schematically described in Supplementary Fig. 15. DUT resistance is always assessed at the read-out voltage of 0.2 V.

Supplementary References

[1] J. Xing, A. Serb, A. Khiat, R. Berdan, H. Xu, and T. Prodromakis. An FPGA-based instrument for en-masse RRAM characterization with ns pulsing resolution. *IEEE Transactions on Circuits and Systems I: Regular Papers*, PP(99):1–9, 2016.

[2] A. Serb, A. Khiat, and T. Prodromakis. An RRAM biasing parameter optimizer. *Electron Devices, IEEE Transactions on*, 62(11):3685–3691, Nov 2015.

[3] Rainer Waser and Masakazu Aono. Nanoionics-based resistive switching memories. *Nature materials*, 6(11):833–840, 2007.

[4] Kyung Min Kim, Doo Seok Jeong, and Cheol Seong Hwang. Nanofilamentary resistive switching in binary oxide system; a review on the present status and outlook. *Nanotechnology*, 22(25):254002, 2011.

[5] J Joshua Yang, Matthew D Pickett, Xuema Li, Douglas AA Ohlberg, Duncan R Stewart, and R Stanley Williams. Memristive switching mechanism for metal/oxide/metal nanodevices. *Nature nanotechnology*, 3(7):429–433, 2008.

[6] Hisashi Shima, Ni Zhong, and Hiro Akinaga. Switchable rectifier built with pt/tiox/pt trilayer. *Applied Physics Letters*, 94(8):2905, 2009.

[7] I. Gupta, A. Serb, R. Berdan, A. Khiat, A. Regoutz, and T. Prodromakis. A cell classifier for RRAM process development. *Circuits and Systems II: Express Briefs, IEEE Transactions on*, 62(7):676–680, July 2015.

[8] Ilia Valov, Eike Linn, Stefan Tappertzhofen, Sebastian Schmelzer, J Van den Hurk, Florian Lentz, and Rainer Waser. Nanobatteries in redox-based resistive switches require extension of memristor theory. *Nature communications*, 4:1771, 2013.

[9] Radu Berdan, Alexander Serb, Ali Khiat, Anna Regoutz, Christos Papavassiliou, and Themis Prodromakis. A-μ-controller-based system for interfacing selectorless RRAM crossbar arrays. *Electron Devices, IEEE Transactions on*, 62(7):2190–2196, 2015.
